# Pediatric schwannoma of the hypoglossal nerve: a case report and narrative literature overview

**DOI:** 10.3389/fonc.2024.1400335

**Published:** 2024-05-28

**Authors:** Elena Sofia Marcandella, Alessandro Boscarelli, Flora-Maria Murru, Giuseppe Abbracciavento, Peter Spazzapan, Jurgen Schleef

**Affiliations:** ^1^ Pediatric Surgery Division, Women’s and Children’s Health Department, University of Padua, Padua, Italy; ^2^ Department of Pediatric Surgery and Urology, Institute of Maternal and Child Health - Istituto di Ricovero e Cura a Carattere Scientifico (IRCCS) “Burlo Garofolo”, Trieste, Italy; ^3^ Radiology Department, Institute for Maternal and Child Health - Istituto di Ricovero e Cura a Carattere Scientifico (IRCCS) “Burlo Garofolo”, Trieste, Italy; ^4^ Department of Pediatrics, Institute of Maternal and Child Health-Istituto di Ricovero e Cura a Carattere Scientifico (IRCCS) “Burlo Garofolo”, Trieste, Italy; ^5^ Department of Neurosurgery, University Medical Centre Ljubljana, Ljubljana, Slovenia

**Keywords:** pediatric schwannoma, hypoglossal schwannoma, tongue disease, neck masses, management

## Abstract

Schwannomas are benign, slow-growing tumors originating from the Schwann cells of nerve sheaths. Extracranial schwannomas are rare, particularly in pediatric populations. Here, we report the case of a hypoglossal schwannoma in a 15-year-old male who experienced tongue paresthesia and fasciculations and difficulty swallowing two years before hospital admission. Magnetic resonance imaging showed an oval mass with sharp and regular limits of approximately 45 × 29 × 25 mm in the cranial portion of the right carotid adipose space, caudal to the right carotid and lateral foramen. The patient underwent surgery, and a histological examination confirmed a schwannoma of the hypoglossal nerve. Six months after surgery, the patient was symptom-free. The literature on schwannomas of the hypoglossal nerve is scarce, with only a few previously reported cases in the adult population. Despite their rarity, schwannomas should be considered in the differential diagnosis of masses located in the neck that present with lingual and occasionally auditory symptoms, even in pediatric patients. Surgical resection is recommended and has a low risk of long-term recurrence.

## 1 Introduction

Schwannomas are non-malignant, solitary, slow-growing neural sheath tumors that affect cranial, sympathetic, and peripheral nerves. About 25–45% of extracranial schwannomas arise in the head and neck. Schwannomas tend to occur in the parotid gland, nose, paranasal sinuses, and oral cavity, but they occur rarely in other locations. Schwannomas originating from the descending loop of the hypoglossal nerve are particularly rare (1, 2). Since De Martel and colleagues first described a hypoglossal schwannoma in 1933, only a few case reports of extracranial schwannomas have been described. The most common location of an extracranial hypoglossal schwannoma is in the parapharyngeal space of older women, and these schwannomas can mimic paragangliomas (2, 3). In 1984, Kaye et al. (4) proposed a classification based on the location of the schwannoma: intracranial (Type A), intracranial/extracranial (Type B), or extracranial (Type C). Signs and symptoms of the tumor are variable and depend on its location, although tongue disturbances are present in many patients (38% of cases) (4). Larger tumors may additionally present with cerebellar and brainstem signs. Management of these tumors typically involves surgical removal. However, surgical resection can be challenging, given the need for an intricate approach to the skull base (5).

To the best of our knowledge, no pediatric cases of hypoglossal schwannoma have been described to date. Here, we present an extremely rare case of a pediatric schwannoma arising from the descending loop of the hypoglossal nerve.

## 2 Case presentation

A 15-year-old male patient presented to our surgical department with a two-year history of paresthesia and fasciculations of the tongue and difficulty swallowing. He also reported that the fasciculations sometimes resulted in impairments in the tone of his language that were consistent with right hemisphere damage, with consequent problems speaking. The patient also reported tinnitus in the right ear within the past year, with sporadic weekly or monthly episodes lasting a few minutes before spontaneously resolving. A neurological examination showed fasciculations on the right side of the tongue. The patient also reported self-perceived, limited rightward tongue mobility. This was confirmed with the observation of reduced external tongue movements. A slight deviation of the tongue to the left was also observed. A magnetic resonance imaging (MRI) study of the neck using gadolinium was performed according to T1 and T2 multiplanar sequences, with suppression of the adipose tissue signal, T2 fast field echo, diffusion-weighted imaging, and Dixon T1 and T2 before and after endovenous administration of paramagnetic contrast.

**Figure 1:**
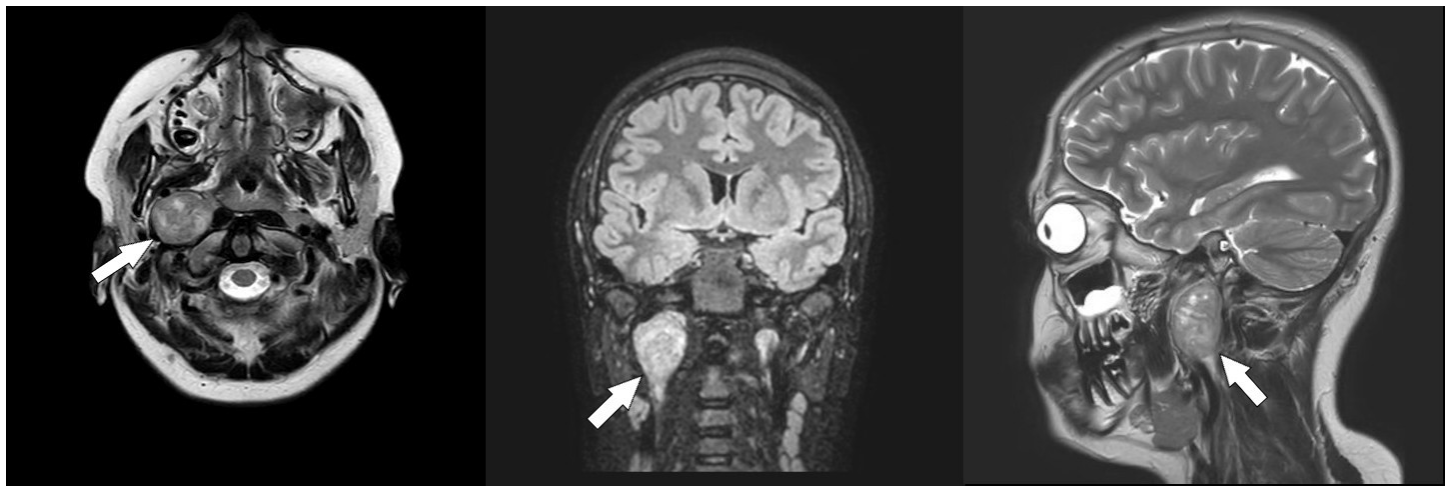
Pre-operative Magnetic Resonance Imaging (MRI). Axial T2-weighted (*left*), coronal fluid-attenuated inversion recovery (FLAIR) (*middle*), and sagittal T2-weighted (*right*) scans showing a neoformation arising from the hypoglossal nerve (*white arrows*).

**Figure 2:**
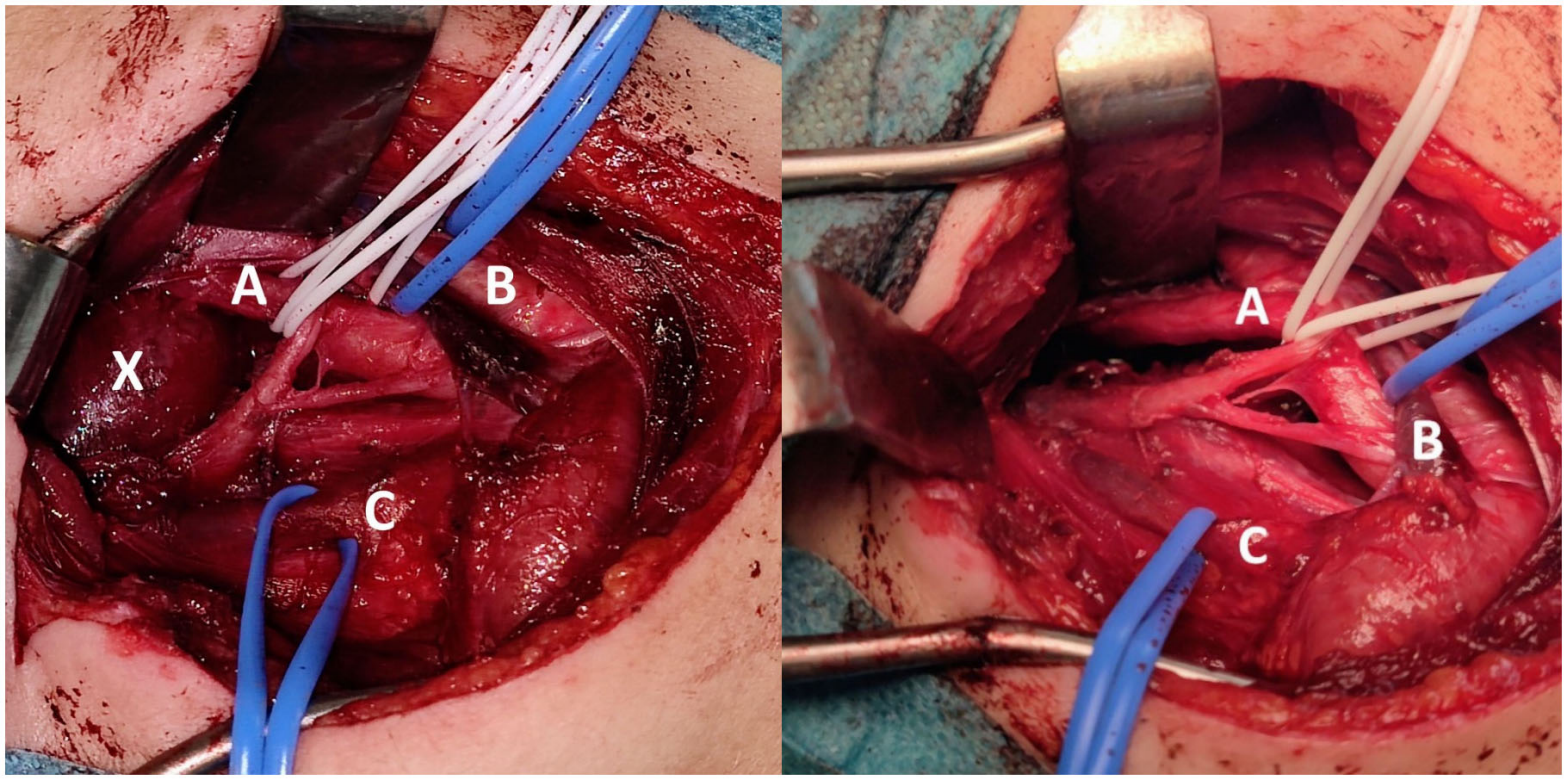
Intraoperative Images. Intraoperative close-up view before (*left*) and after (*right*) tumor excision through an oblique incision at the level of the right sternocleidomastoid muscle in the supine position (*A = carotid artery, B = jugular vein, C = nerve bundle, X = tumor*).

**Figure 3:**
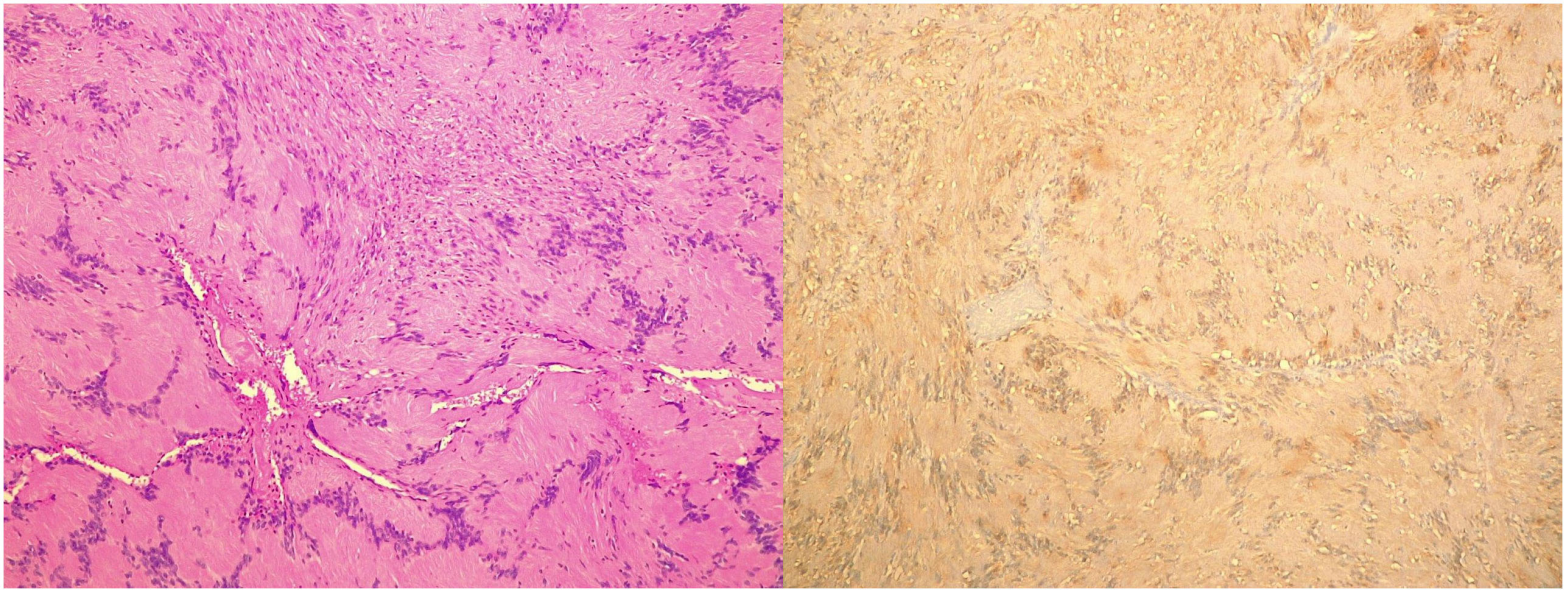
Histopathological Images. Microscopic image (*left*) and S100 positivity image (*right*) of a hypoglossal schwannoma taken through a 10× objective on hematoxylin and eosin–stained histological sections.

**Figure 4:**
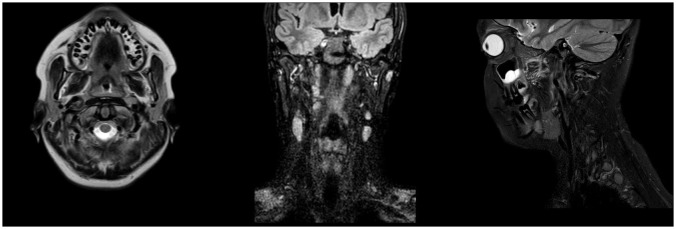
Post-operative Magnetic Resonance Imaging (MRI). Axial T2-weighted (left), coronal FLAIR (middle), and sagittal T2-weighted (right) post-operative MRI scans demonstrating complete excision of the hypoglossal schwannoma at 6-month follow-up.

An oval mass with sharp and regular limits was detected at the most cranial portion of the right carotid adipose space, caudal to the right carotid and lateral foramen. The mass was approximately 45 × 29 × 25 mm and was characterized by inhomogeneous T2 hyperintensity due to some contextual hyperintense areas in T2 (**Figure 1**). The mass displaced the internal carotid artery anterolaterally and exerted modest compression posterior to the internal jugular vein, with a slight increase in the caliber of the upstream sigmoid sinus. At this point, the differential diagnosis was either a schwannoma of the hypoglossus or a vagus nerve/hypoglossal paraganglioma. The MRI scans of the brain uncovered no pathological findings. Oncologists were consulted, and they recommended testing urinary vanillylmandelic acid and homovanillic acid levels for a complete oncological framing; these showed positive results. Preoperative angio–computed tomography confirmed the hypothesis of an expansive lesion of the nerve sheath. Under general anesthesia, the patient underwent surgical removal of the mass via an oblique incision at the level of the right sternocleidomastoid muscle in the supine position. The portion of the tumor posterior to the internal carotid artery and anterior to the internal jugular vein was adequately identified (**Figure 2**, left). The mass was then isolated by carefully preserving the nerve structures and monitoring them with an evoked potential monitoring system. Excision was macroscopically completed (**Figure 2**, right). Motor potentials were unaffected after surgery. A Redon-type suction drain was placed and then removed after two days, and the patient was discharged on the fifth postoperative day. Antibiotic therapy with ampicillin/sulbactam and steroidal therapy was administered for seven days. This was initially administered intravenously and then orally. A histological examination confirmed the radiological suspicion of a schwannoma, as the morphophysiopathological picture was consistent with a schwannoma originating from the hypoglossal nerve (S100+, calretinin+, SOX10+, D240+, and Ki67 5%) (**Figure 3**). After removing the mass, the patient no longer complained of lingual discomfort but experienced mild eyelid ptosis, miosis, and paresthesia in the ipsilateral cheek, which steadily improved. Although a six-month follow-up period is relatively short for investigating possible relapse in such a pediatric case, there were no signs of tumor recurrence at a follow-up MRI performed six months after surgery (**Figure 4**), and the patient was in excellent general condition.

The main characteristics of the present case are summarized in **Table 1**.

**Table 1:** Main details of the case of schwannoma of the hypoglossal nerve described in this article.

Age (years)	Sex	Comorbidity	Symptoms	Positive markers	Diagnostic imaging	Tumor localization	Tumor size (mm)	Treatment	Histology	Follow-up
15	M	None	- Paresthesia; fasciculations of the tongue - Difficulties in swallowing - Increased right hemi-language tone - Pain in the posterior cervical region - Tinnitus	- UOA + - UVA +	- MRI - angio-CT	Right hypoglossal nerve	45x29x25	Open removal	- S100 + - Calretinin + - SOX 10 + - D240 + - Ki67 5%	- Clinical - MRI every 6 months

mm, millimetres; M, Male; UOA, Urinary Omovanillic Acid; UVA, Urinary Vanilmandelic Acid; MRI, Magneti Resonance Imagin; CT, Computed Tomography.

## 3 Discussion

Schwannomas are benign tumors even if a progression to malignancy can be observed. They usually develop from the sensory cranial nerves, most commonly the eighth and tenth nerves (the acoustic and vagus). Schwannomas of the hypoglossal nerve (12th cranial nerve) are rare, as this is a purely motor nerve (1, 4, 6, 7). However, schwannomas arising from this nerve are usually associated with hemiatry, fasciculations, and tongue deviation during protrusion, which can (rarely) also be caused by schwannomas of the cervical vagus nerve. Given the absence of functional action of the descending branch of the hypoglossal nerve, schwannomas in this location should be neurologically asymptomatic as long as there is no compression of the adjacent cranial nerves. In our case, a medical history and detailed description of the symptoms provided by the patient played a fundamental role in suspecting a disease related to the hypoglossal nerve. This highlights the importance of listening to patients report their symptoms even if macroscopic signs are not evident during a medical examination. If a hypoglossal nerve schwannoma is suspected, a true contrast MRI is necessary to obtain evidence of a well-limited mass surrounding or including the nerve. Biological markers can also be tested to better define the diagnosis. Curative resection aims to relieve symptoms, but it is also necessary because both primary and recurrent schwannomas can show progression to sarcomas (3, 5–8).

Based on this case, we suggest gaining standard surgical access through a pre-sternocleidomastoid incision and carefully monitoring evoked potentials. We strongly recommend nerve monitoring in particular to reduce the risk of permanent injury to sensory and motor nerve functions (5, 9). Our case provides further evidence of favorable outcomes following complete surgical excision of hypoglossal schwannomas. The goal of surgery should be gross total resection of the tumor with preservation of nerve function when feasible. Recovery following surgery tends to be favorable, with many patients experiencing mild and usually well-tolerated neurologic deficits (10–12). Regardless of the nerve from which the schwannoma originates, regular clinical and ultrasonography checkups on a semi-annual basis for at least the first three years after surgery and annually thereafter are strongly recommended to facilitate prompt detection and treatment of any recurrences. However, recurrences should be extremely rare after curative resection (5).

Notably, schwannomas can also occur in other sites in childhood, including the pterygopalatine fossa, oropharynx, larynx, eye socket, sellar region, intracranial, trigeminal, or intramedullary. The symptoms vary according to the site. For tumors arising in the orbital region, the symptoms are referred to as visual spheres, such as binocular vision, strabismus, and proptosis. Remarkably, even for pterygopalatine localization, the main symptom was reduced vision, accompanied by a central dark spot. In the oropharyngeal region, the reported symptoms include sore throat, nasal congestion, voice changes, and odynophagia. For the laryngeal region, the symptoms encountered were voice changes, hemoptysis, snoring, difficulty in breathing, dysphagia, and dry cough. Lastly, with regard to localization in the central nervous system, symptoms appear to be nonspecific, such as nausea and headache or weakness in the limbs. Overall, schwannomas tend to be more frequent in the male than in the female sex. The presentation is well distributed in the first and second decades of life. Notably, none of the cited cases had a direct association with neurofibromatosis, thus confirming that schwannomas in pediatric patients can be an isolated finding. According to the literature, complete resection is the treatment of choice, with variations in the surgical approach. Outcomes are generally good, with no recurrence if complete resection can be achieved (13–21). The main details of pediatric schwannomas arising from different sites beyond the hypoglossal nerve are reported in **Table 2**.

**Table 2:** Literature overview of pediatric schwannomas cases.

Authors, Year	Age (yrs)	Sex	Comorbidity	Symptoms	Pre-op Markers	Imaging	Tumor localization	Tumor size (mm)	Treatment	Histology	Outcomes
Cai H et al., 2024 (13)	6	M	None	Reduced vision in the left eye	Not reported	- CT - MRI	Left PPF	45×33×40	Intranasal endoscopic removal	- S100+ - SOX10+ - Ki67 <5%	- Regained full vision - No recurrence
Rosi-Schumacher M et al., 2023 (14)	14	M	None	- Sore throat - Nasal congestion - Expanding mass in the back of the mouth - Voice changes - Odynophagia	Not reported	CT	Oropharyngeal	26x39x53	Open excision	- S100+ - SOX10+	Not reported
Hamam YA et al., 2023 (15)	12	M	None	- Hemoptysis - Snoring - Difficulty of breathing - Dysphagia - Fatigue - Dry cough - Voice changes	Not reported	CT	Midline supraglottic mass extending onto sclerotic arytenoids	40×30	Endoscopicexcision	S100 +	- No symptoms - No recurrence
Shao Y, 2023 (16)	12	M	Family history of NF	Binocular vision	Not reported	- CT - MRI	Posterior extraconal compartment of the left eye	Not reported	Left fronto-temporal craniotomy	- S100 + - SOX10 + - Ki67 <1%	No recurrence
Sadiqo R et al., 2022 (17)	1	F	None	- Severe visual impairments in both eyes - Strabismus - weakness	Elevate FT3 levels	MRI	Sellar region extending onto suprasellar region, third ventricle & hypophysis	40x35x30	Transcranic approach	- S100 + - GFAP - - EMA -	- Regained full vision - No recurrence
Colas Q et al., 2022 (18)	8	M	None	Progressive, painless and non-inflammatory proptosis of the right eye	Not reported	- CT - MRI	Right extraconical localization with ethmoidal extension	33x25x28	Transcaruncular approach	- S100 + - SOX10 + - SMA -	No recurrence
Hirokawa D et al., 2019 (19)	9	M	None	- Headache - Nausea	Not reported	MRI	Left cerebello-medullary fissure	Not reported	Left lateral sub-occipital craniotomy	- S100 + - GFAP - - Oligo2 - - Ki47 4/5%	- No symptoms - No recurrence
O’Connor KP et al., 2019 (20)	14	M	None	Diplopia	Not reported	MRI	Posterior fossa along the cisternal segment of the trigeminal nerve extending into the cavernous sinus	Not reported	Pterional craniotomy	S100 +	No recurrence
Wang K et al., 2018 (21)	9	M	-Syringomyelia - Right thoracolumbar scoliosis	Lower extremities weakness	Not reported	MRI	Intramedullary lesion extending at the level of T8 vertebral body	Not reported	Laminectomy T7-T8	Not reported	Progressive postoperative spinal kyphosis observed 3 years after surgery

yrs, years; Pre-op, pre-operative; mm, millimetres; M, male; F, female; NF, neurofibromatosis; CT, computed tomography; MRI, magnetic resonance imaging; PPF, pterygopalatine fossa.

## 4 Conclusions

Extracranial hypoglossal schwannomas are quite rare. However, schwannomas should be considered in the differential diagnosis of masses in the neck that present with lingual and sometimes auditory symptoms, even in pediatric patients. Surgical resection is recommended, is often well tolerated, and has a low risk of long-term recurrence. According to our experience and current evidence, the outcomes after complete surgical excision of hypoglossal schwannomas are overall favorable.

## Data availability statement

The raw data supporting the conclusions of this article will be made available by the authors, without undue reservation.

## Ethics statement

The requirement of ethical approval was waived by Institute for Maternal and Child Health IRCCS Burlo Garofolo for the studies involving humans because retrospective nature of this report. The studies were conducted in accordance with the local legislation and institutional requirements. Written informed consent for participation in this study was provided by the participants’ legal guardians/next of kin. Written informed consent was obtained from the individual(s) for the publication of any potentially identifiable images or data included in this article.

## Author contributions

EM: Conceptualization, Data curation, Investigation, Writing – original draft. AB: Data curation, Investigation, Methodology, Writing – original draft, Writing – review & editing. F-MM: Data curation, Writing – original draft. GA: Data curation, Writing – original draft. PS: Methodology, Writing – review & editing. JS: Conceptualization, Methodology, Writing – review & editing.
